# Enabling Synthetically Feasible Molecular Editing in Drug Discovery via Reaction-Regulated Graph-Based Genetic Algorithms

**DOI:** 10.1021/jacsau.6c00094

**Published:** 2026-03-23

**Authors:** Sung Wook Moon, Se Hwan Ahn, Jin Hee Ahn, Hyun Woo Kim

**Affiliations:** † Department of Chemistry, 65419 Gwangju Institute of Science and Technology, Gwangju 61005, Republic of Korea; ‡ JD Bioscience Inc., Gwangju 61011, Republic of Korea

**Keywords:** cheminformatics, drug discovery, molecular design, synthetic feasibility, genetic algorithm, reaction rules

## Abstract

Machine learning-based generative models have enabled the efficient and accurate exploration of vast chemical space in recent decades. However, many approaches do not explicitly account for the synthetic feasibility of generated molecules due to the challenge of integrating both theoretical and experimental perspectives. To overcome this challenge, here we propose a reaction-regulated graph-based genetic algorithm, namely R^2^GB-GA, that enables synthetically feasible molecular editing through effectively exploring complex chemical space for molecular design. By embedding domain-specific reaction rules into the evolutionary process, our method enables site-selective molecular modifications to a scaffold that allows for both preserving specific scaffolds and changing only specific molecular substructures. We show that the proposed method generates more synthetically accessible molecules across diverse generative tasks, outperforming conventional approaches when evaluated using fragment-based and pathway-based scoring methods. In addition, we illustrate the practical applicability of our method to drug design through ligand design targeting the inhibition of heat shock protein 90, a representative chaperone protein in cancer therapy. We believe that with the use of a reaction-based framework, our approach could be applied as a general method for a broad range of drug discovery strategies including late-stage functionalization.

## Graphical abstract

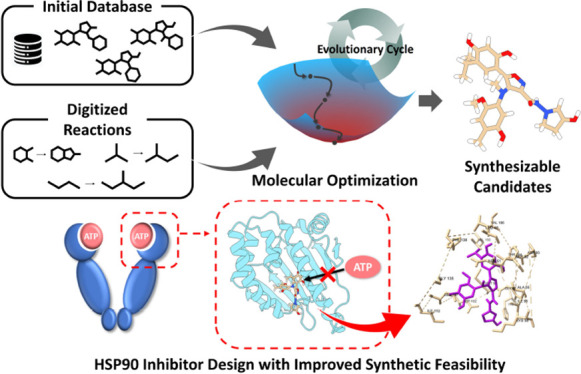

## 1 Introduction

Designing novel molecules plays a crucial role in the history of chemistry. From the discovery of drug candidates^1–4^ to advanced materials,^5–8^ molecular design has shown its potential for broad applications across diverse fields, underscoring its fundamental importance and guiding the direction of most areas of scientific research. Although numerous attempts have been made through trial-and-error experiments and rational design approaches, the emergence of machine learning (ML) as a subset of artificial intelligence (AI) has garnered significant attention as a game changer, now tightly embedded within the framework of molecular discovery.^9,10^

In recent decades, numerous generative models have been developed, enabling the creation of novel molecules and enhancing the exploration of chemical space. This exploration of chemical space can be regarded as an “optimization” algorithm,^11,12^ which proceeds step by step to identify molecules that satisfy the desired properties. In general, when continuous and differentiable objective functions are available, methods based on deep learning architectures are widely employed for the rapid and efficient exploration of chemical space.^13–15^ However, in cases involving discontinuous or nondifferentiable objective functions, stochastic approaches offer an alternative, frequently resulting in improved efficiency and interpretability. There are various types of stochastic approaches such as evolutionary algorithms,^16^ Monte Carlo tree searches,^17^ and swarm intelligence.^18^ In this study, we employ the genetic algorithm (GA),^19^ a representative method within evolutionary algorithms inspired by the principle of natural selection. Although various types of GA have been proposed, the graph-based genetic algorithm (GB-GA)^20^ has been widely adopted because of its effectiveness in capturing molecular structures and preventing invalid chemical structures.

Beyond developing faster and more accurate molecular design models, synergistic collaboration with experimental research is increasingly recognized as essential for molecular discovery. The primary goal of molecular discovery is to identify molecules with practical applications, making it crucial to address the limitations of purely theoretical work. Consequently, establishing a robust feedback loop between theoretical and experimental studies has become particularly important.^21–24^ A key challenge in this workflow is that molecules proposed by these generative models are often difficult to synthesize in practice.^25–27^ To propose molecules with desired properties, most generative models do not directly synthesize molecules but instead perform structural perturbations on molecular scaffolds. Thus, these models may lead to a discrepancy between the computationally designed molecules and their practical feasibility.

To bridge the gap between computational designs and experimental validations, previous studies have introduced several scoring functions, such as SAScore,^28^ SCScore,^29^ RAscore,^30^ and DFRscore.^31^ While these scoring functions are useful for virtual screening, their direct application in molecule generation is limited. They often fail to accurately reflect real-world synthetic feasibility and, as a result, cannot reliably guide the optimization of generated molecules toward desired practical features. Hence, we introduce a strategy to enhance synthetic accessibility throughout the molecular generative model by applying realistic, reaction-regulated structural perturbations in order to overcome this limitation.

Compared with other approaches that incorporate synthetic accessibility, a commonly used strategy is post hoc scoring, in which molecules are first generated and then filtered by a synthesizability metric.^25^ However, because generation precedes feasibility assessment, post hoc scoring can be inefficient during optimization, as it repeatedly discards generated molecules.^32^ Moreover, even if such metrics can indicate whether a generated molecule is synthesizable, they typically do not provide an explicit synthetic route.^33^ Explicit synthesis planning^34,35^ is one approach to address this limitation by providing synthetic routes for generated molecules. However, the associated computational cost makes it challenging to incorporate into the optimization loop.^36^ In contrast, our reaction-based strategy explicitly considers synthetic feasibility during molecule generation, while enabling the proposal of plausible synthetic routes at a manageable computational cost. Furthermore, our method not only addresses the limitations above but also offers several additional advantages. For example, by controlling the reactions employed, our method can preserve a specific scaffold while selectively modifying only the desired substructures, making it particularly well suited for applications such as late-stage functionalization.^37^

The reaction-regulated structural perturbations were implemented by augmenting the mutation operator in the GB-GA to mimic transformations between reactants and products in real chemical reactions, thereby aligning the generative process more closely with experimental feasibility.

This article is organized as follows. First, we demonstrate that our algorithm generates molecules with higher synthetic feasibility by evaluating it across multiple databases. Next, we validate the effectiveness of our approach through a rediscovery task. Finally, we apply our algorithm to design inhibitors targeting the heat shock protein 90 (HSP90), a chaperone protein implicated in cancer progression.^38–41^

## 2 Method

### 2.1 Reaction-Regulated Graph-Based Genetic Algorithm

Figure 1 provides the difference between the conventional approach and our reaction-based scheme. Our R^2^GB-GA broadly follows the similar fundamental steps consisting of selection, crossover, and mutation steps. In the selection step, high-scoring molecules are retained, whereas crossover and mutation introduce structural modifications, thereby propagating molecular diversity throughout the population. The conventional GB-GA mutation operator (Figure 1c, left) operates on molecular graphs, ensuring the generation of chemically valid structures. However, since it generates structures by randomly adding or modifying atoms and bonds, the resulting molecules may be chemically valid but are not necessarily synthetically feasible. For instance, the conventional scheme often allows substitution of nitrogen with carbon at various atomic positions owing to valence electron difference between carbon and nitrogen. In a practical case, however, achieving site-selectivity in long chain or cyclic structures remains a major challenge, typically requiring sophisticated catalytic systems or other advanced synthetic approaches.^42^ In contrast, the reaction-driven mutation operator employed in this work (Figure 1c, right) identifies chemically reactive sites within the molecular population according to a set of predefined reaction rules. By constraining mutations to these chemically feasible sites, this strategy is designed to enhance the generation of synthetically accessible molecules compared to conventional methods.

**Figure 1:**
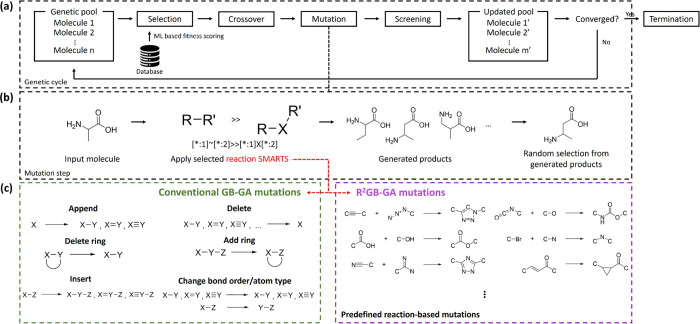
Overall schematic of the R^2^GB-GA and its differences from the conventional GB-GA. (a) Overall workflow of the GA framework. (b) Schematic representation of the mutation process in GB-GA. (c) Mutation operations in conventional GB-GA (green box) and predefined reaction-driven operations employed in R^2^GB-GA (purple box).

The R^2^GB-GA framework remains largely consistent with the conventional GB-GA scheme, except for the mutation operator. In the selection step, molecules for the next generation were chosen using ML-based fitness scoring, for which a Gaussian process regression (GPR) model was employed. The crossover operator of the GA was adapted from the implementation by Jensen.^43,44^ The reaction database employed for mutation was constructed from the public database provided by Datamol,^45^ with a total 362 reactions. Molecular representations were generated as extended-connectivity fingerprints (ECFP4)^46^ using the RDKit software.^47^ The initial geometries of molecules were obtained by converting SMILES strings with OpenBabel.^48^ Docking simulations to evaluate the binding affinity of the generated candidate molecules were conducted with GalaxyDock-DL^49^ and AutoDock Vina.^50,51^

## 3 Results and Discussion

### 3.1 Benchmarking Synthetic Feasibility

First, we evaluated the performance of our R^2^GB-GA using various synthetic feasibility scores, which demonstrated its effectiveness in generating more accessible molecules compared to conventional methods. To assess the synthetic feasibility of the molecules generated by our R^2^GB-GA, we performed an evaluation using three distinct scoring methods: SAScore,^28^ SYBA,^52^ and RAscore.^30^ In addition, to demonstrate the broader applicability of our method in enhancing synthetic accessibility, we applied the generative model to multiple molecular databases. As shown in Figure 2, we evaluated our method on three generative tasks. First, we assessed its performance by applying our structural perturbation operators (crossover and mutation), starting from the ChEMBL database^53^ (Figure 2a). Here, a molecular pool consisting of 2000 molecules was propagated. Next, based on the QM8 database,^54,55^ a pool of 500 molecules was generated to target an excitation energy of 6.2 eV (Figure 2b). Finally, for the design of HSP90 inhibitors, a pool of 400 molecules was propagated to obtain inhibitors with low IC_50_ values (Figure 2c). Details of the HSP90 inhibitor design will be discussed in a later section. The variation in pool sizes of three tasks arises from differences in the size of the databases. In all cases, the molecules were propagated for 10 generations, and the resulting molecules were evaluated using three methods for scoring synthetic accessibility.

**Figure 2:**
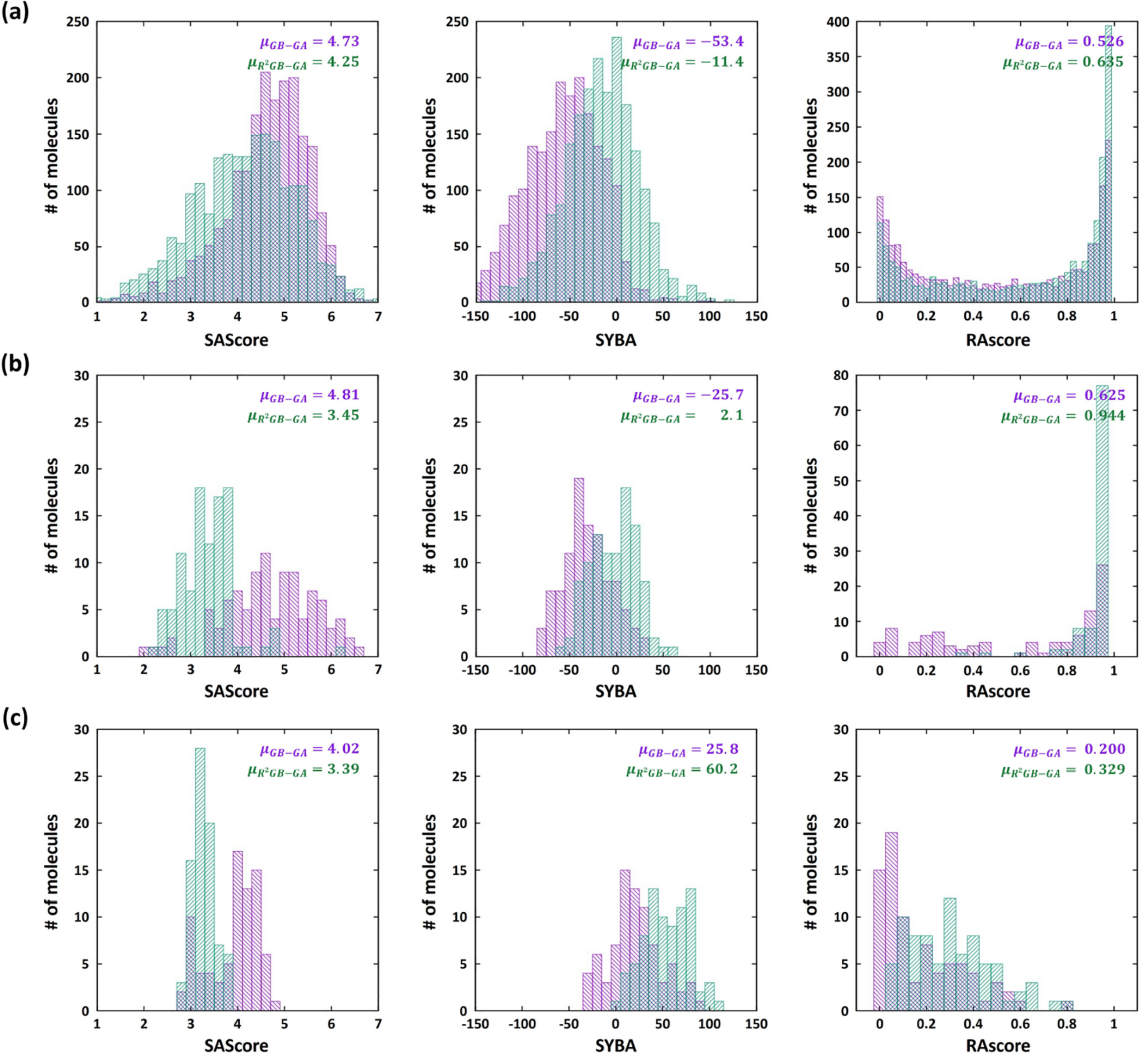
Comparison of synthetic feasibility metrics for molecules generated using the conventional GB-GA and R^2^GB-GA. (a) Generation of molecules from the ChEMBL database, (b) design of molecules based on the QM8 data set, and (c) ligand design for the HSP90 inhibitor. Distributions for the conventional GB-GA (purple) and R^2^GB-GA (green) are depicted.

When examining the overall tendency, it can be observed that the R^2^GB-GA results (highlighted in green) consistently outperform the conventional GB-GA (highlighted in purple) across all cases. A lower SAScore value and higher SYBA and RAscore values indicate greater synthetic accessibility. The fact that all three metrics show favorable values across the three tasks suggests that our method provides a clear advantage in generating synthetically accessible molecules compared to the conventional approach.

When evaluated on the ChEMBL data set, conventional GB-GA without the reaction-driven scheme yielded average scores of 4.73, −53.4, and 0.526 for SAScore, SYBA, and RAscore, respectively. In contrast, the reaction-driven scheme achieved improved values of 4.25, −11.4, and 0.635. Notably, the number of molecules with RAscore values approaching 1 nearly doubled after applying the R^2^GB-GA. This is likely because RAscore evaluates accessibility based on reaction pathways, unlike SAScore and SYBA, which estimate synthetic accessibility through fragment-based scoring. Since R^2^GB-GA generates molecules by mimicking real chemical reactions, it is not surprising that a larger proportion of molecules achieve higher scores in RAscore, which evaluates synthetic accessibility based on reaction pathways. The results obtained from the QM8 data set exhibit a similar trend to those observed for the ChEMBL database. The average SAScore, SYBA, and RAscore values of 4.81, −25.7, and 0.625 obtained from the conventional GB-GA improved to 3.45, 2.1, and 0.944, respectively, when applying the R^2^GB-GA.

For the HSP90 inhibitor generation task, higher scores were consistently obtained across all scoring metrics, as observed in the previous cases. Specifically, the average SAScore, SYBA, and RAscore improved from 4.02, 25.8, and 0.200 to 3.39, 60.2, and 0.329, respectively, when using the R^2^GB-GA. However, unlike the fragment based metrics like SAScore and SYBA, the RAscore displayed a distinct behavior. While the application of R^2^GB-GA to the ChEMBL and QM8 data sets resulted in a notable increase in molecules with RAscore values close to 1, the HSP90 inhibitor task showed a shifted histogram distribution. This can be attributed to the fact that the generation of the HSP90 inhibitor was carried out with the fixed core functional scaffold, which directly influences the interactions with the target protein. Unlike the previous two cases without initial structural constraints, the fixed scaffold in this task makes it more difficult to define clear reaction pathways, which likely led to the observed results. It is well-known that reaction pathway-based scoring methods exhibit an inherent dependency on molecular size,^52,56^ and thus these results are not surprising. In addition, because RAscore is a reaction-based metric, its evaluation may be biased if the reaction templates included in the generative model substantially overlap with those used to calculate RAscore, potentially leading to an overestimation of synthetic accessibility. To address this issue, we excluded the reaction templates that overlap with those used in the RAscore evaluation from the reaction set employed in R^2^GB-GA and repeated the three generation tasks (Figure S3). As shown in the figure, R^2^GB-GA model still generates molecules with high RAscores, suggesting that our results are unlikely to be driven by overlap between reaction templates.

Although different trends are observed across individual tasks, it is evident that R^2^GB-GA consistently produces molecules with higher synthetic accessibility compared to the conventional GB-GA. This result suggests that our method is more effective in generating molecules that are more likely to be validated in practical experiments.

### 3.2 Goal-Directed Benchmarking

The advantages of the R^2^GB-GA include not only the generation of synthetically feasible molecules but also higher efficiency in exploring chemical space compared with conventional approaches. In this section, we evaluate the R^2^GB-GA for exploring chemical space using the goal-directed benchmarks provided in GuacaMol.^57^ Specifically, we assessed its performance through the rediscovery of three drug-like molecules, namely celecoxib, troglitazone, and thiothixene, as shown in Figure 3a. For the initial database, we used the postprocessed ChEMBL24 data set^58^ for the rediscovery task. The postprocessing procedure involved removing molecules that exhibited a Tanimoto similarity greater than 0.323 to the target molecules, in order to exclude near-identical structures and ensure that the rediscovery task served as a valid performance benchmark. The rediscovery task was performed based on the Tanimoto similarity between the target molecule and the generated molecules, and 100 optimization trajectories were conducted for each method to obtain statistically reliable results.

**Figure 3:**
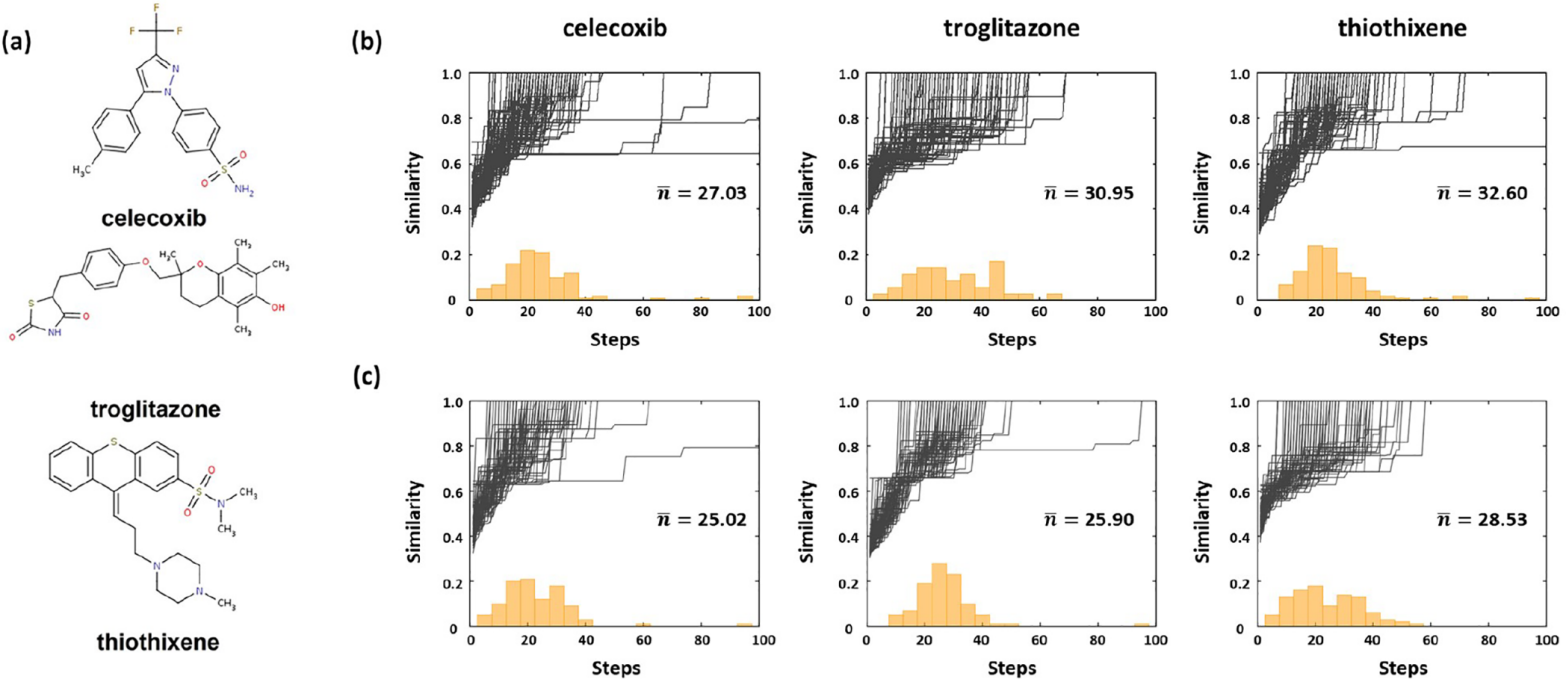
(a) Structures of three drug-like molecules used in the rediscovery task. (b) Optimization trajectories of maximum Tanimoto similarity over steps for each target molecule with the conventional GB-GA, and (c) with the proposed R^2^GB-GA. The orange histograms show the distribution of steps at which the target molecule is discovered. The *n*- denotes the average number of steps to find the target molecule.

Before performing the benchmark tasks, we first evaluated the sensitivity of R^2^GB-GA to the size of the reaction pool. Since structural modifications in R^2^GB-GA are generated via reaction-based operator, the size of the reaction set can substantially influence the diversity of accessible structures. To assess whether our reaction pool is sufficiently large, we utilized the United States Patent and Trademark Office (USPTO) reaction data set^59^ for reactions that can be mapped to our reaction pool. We found that approximately 10^6^ reactions can be matched to our reaction set, suggesting that the reaction set used provides substantial coverage of the data set. We then tested randomly sampled reaction subsets to quantify how performance varies with the size of the reaction pool. Details of the subset construction are provided in the Supporting Information S3. As a result, rediscovery performance depended on the reaction pool size, with smaller pools generally yielding lower performance (Figure S4). Beyond a certain pool size (approximately 50% of the full reaction pool), performance became less sensitive to further increases in the number of templates. These results indicate that using a sufficiently large reaction pool is important for the robust performance of R^2^GB-GA.

In the goal-directed benchmark involving three drug-like molecules, the rediscovery trajectories can be found in Figure 3b,c. When comparing the optimization trajectories without R^2^GB-GA (Figure 3b) and with R^2^GB-GA (Figure 3c), we found that R^2^GB-GA rediscovered the target molecule more efficiently even though both were evaluated under identical conditions. For the rediscovery of celecoxib, troglitazone, and thiothixene using the conventional GB-GA, the average numbers of steps were 27.03, 30.95, and 32.60, respectively. In contrast, the R^2^GB-GA rediscovered the target molecules in 25.02, 25.90, and 28.53 steps, respectively. From the rediscovery task, the R^2^GB-GA exhibits approximately 10% higher efficiency compared with conventional methods. This enhanced efficiency can be attributed to a reaction-driven method, which allows larger and chemically meaningful structural modifications in each generation, thereby accelerating exploration of relevant subspaces within the chemical space.

### 3.3 Ligand Design for HSP90 Inhibition

Finally, we applied our R^2^GB-GA framework to design realistic candidate molecules for HSP90 inhibitors. We focus on the design of ligands that exhibit inhibitory activity against the HSP90 protein, a molecular chaperone that supports the survival of cancer tissue.^38–40^ HSP90 has four isoforms, HSP90α, HSP90β, GRP94, and TRAP1, which are structurally similar but are differentially overexpressed depending on the types of tumors.^60^ Here, we focus on HSP90β, which is frequently found to be overexpressed in lung tumors.^61,62^ The chaperone activity of HSP90 is driven by ATP binding and consequent hydrolysis.

Accordingly, we focused on the design of competitive inhibitors that occupy the ATP-binding pocket in the N-terminal domain of HSP90, thereby emphasizing the therapeutic potential of HSP90 inhibition in cancer treatment. To facilitate effective binding of the ligand within the pocket, we constrained the molecular design around a resorcinol-based core scaffold,^63–65^ enabling systematic and site-selective modification of peripheral substituents as shown in Figure S1. The initial database was constructed from the ChEMBL database.^53,66^ IC_50_ values for human HSP90AB1 (ChEMBL target ID: ChEMBL4303) were retrieved using the following query parameters: Target Type set to “SINGLE PROTEIN” and Organism set to “Homo sapiens”. Based on the range of values present in the IC_50_ database, we set IC_50_ value of 10 nM as a generation target. As described in Section 3.1, a pool of 400 molecules was propagated for 10 rounds of generation.

Before analyzing the generated ligands, we provide additional analysis of the reaction template pool used to generate HSP90 inhibitor candidates. As noted above, our 362 reaction templates were derived from 127 reactions in Datamol^45^ and can be broadly categorized into 101 coupling reactions, 17 functional group interconversions, and 9 substitutions. This reaction set includes transformations frequently used in medicinal chemistry for drug-like substrates, such as Suzuki-Miyaura coupling, Buchwald-Hartwig coupling, and amide bond formation. A reaction frequency analysis (Table S2) indicates that a large fraction of the frequently observed reactions are medicinal chemistry-relevant transformations, with the top ten reactions accounting for 79% of entire reactions. Because this uneven distribution of reaction usage may lead to bias, we assessed the influence of the most frequently used reactions by removing the top 10 reactions in Table S2 and regenerating HSP90 inhibitor candidates. We compared three reaction pools: (1) the full reaction pool, (2) a size-matched control pool obtained by randomly removing 10 reactions, and (3) the pool excluding the top 10 most frequent reactions. As shown in Figure S5, the full pool and the size-matched pool show similar performance. In contrast, excluding the top 10 reactions increases the mean predicted IC_50_ by 0.4 nM after 10 generations, corresponding to one or two additional generation steps to reach comparable potency. Additionally, the CPU time per step increased from 491 to 545 s (an increase of approximately 11%) relative to the two reference pools. These results suggest that although the highest-frequency reactions influence optimization performance, their impact on computational efficiency is more pronounced.

Next, we examined the novelty of the generated molecules. Since one of the primary goals of molecular design is to explore new chemical spaces, it is also important to generate molecules that are distinct from the initial pool. Figure 4 represents novelty of generated molecules. Here, let *D* be the reference set and *G* the set of generated molecules. The novelty of a generated molecule *g* with respect to the reference set *D*, *N*(*g*;*D*), was calculated by

$$N(g;D)=1-\max_{d\in D}(T(\phi (g),\phi (d))),\;g\in G$$ 1

where *T*(·, ·) denotes the Tanimoto similarity between two fingerprints, and ϕ­(*m*) represents the ECFP4 fingerprint vector of molecule *m*. Specifically, for each generated molecule we compute the highest Tanimoto similarity to the molecules in the reference set. Thus, higher *N*(*g*;*D*) values indicate more novel structures, whereas *N*(*g*;*D*) = 0 means that an identical molecule already exists in the reference database.

**Figure 4:**
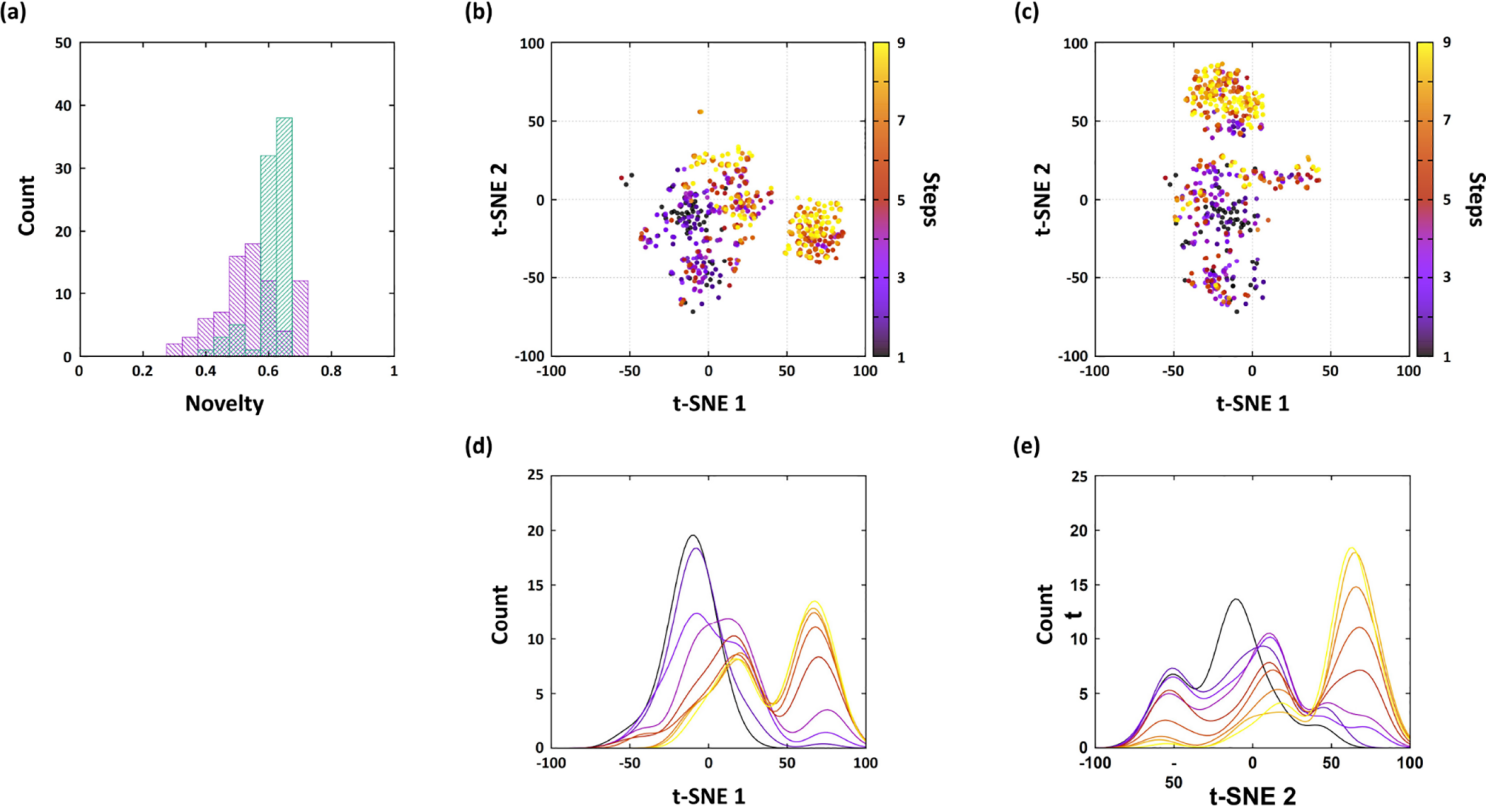
(a) Novelty of generated HSP90 inhibitors for the conventional GB-GA (purple) and R^2^GB-GA (green). (b) Visualization of ECFP4 fingerprints across generation steps during HSP90 inhibitor design for the conventional GB-GA using a t-SNE plot. (c) The corresponding t-SNE visualization for the R^2^GB-GA. Distribution of generated molecules along a t-SNE axis chosen for visualization, shown for the two methods: (d) GB-GA and (e) R^2^GB-GA. Color scheme is the same as in panels (b) and (c).

As shown in Figure 4a, the green distribution from the R^2^GB-GA exhibits a higher average novelty value of 0.437 compared with the purple distribution from the conventional approach of 0.370, indicating that the proposed method generates more novel structures. We attribute the observed difference in novelty to the reaction-based mutation, which modifies molecular structures more extensively than the conventional approach. While the conventional approach introduces structural perturbations through single atom or single bond modifications, our method follows actual chemical reactions, allowing more diverse structural transformations, ranging from the atomic level to the level of entire pharmacophores depending on the reaction type. This behavior indicates that the R^2^GB-GA facilitates the efficient exploration of larger chemical spaces. Meanwhile, some molecules generated by the conventional GB-GA exhibited higher novelty than those generated by R^2^GB-GA. A total of 28 molecules from the conventional GB-GA exhibited novelty values above 0.6, where the R^2^GB-GA molecules were mainly distributed. For these molecules, the average values of the SAScore, SYBA, and RAscore were 3.69, 37.3, and 0.21, respectively, which suggests lower synthetic feasibility than the corresponding averages for R^2^GB-GA (3.39, 60.2, and 0.33). These results indicate that certain molecules from the conventional GB-GA exhibit higher novelty but tend to occupy synthetically impractical regions of chemical space, thereby limiting their applicability in realistic molecular design.

Additionally, we compared our method with the conventional approach by analyzing the generation pathway. Figure 4b,c show the evolution of the generation pool across genetic cycles, visualized by reducing the ECFP4 fingerprints to two dimensions using t-distributed stochastic neighbor embedding (t-SNE).^67^ Although both generative models guide the initial pool toward an optimum region, each converges into distinct region of the chemical space. Indeed, the difference in convergence is not surprising, as the two models apply structural perturbations in distinct mechanisms. Since our method aims to generate molecules with higher synthetic feasibility than those from the conventional direction, such divergence in the convergence pathway is expected. More specifically, the convergence process reveals a notable difference between the two methods. In the conventional approach, an additional cluster emerges after a few steps. During the convergence process toward the optimal region, the population passes through this intermediate cluster, and a fraction of the individuals remains in this cluster even after several steps. This is clearly reflected in the one-dimensional distribution along the t-SNE1 axis shown below Figure 4b, where a portion of the population remains localized near the local minimum around 20 even after several steps. In contrast, the R^2^GB-GA exhibits a significantly faster propagation of the population toward the optimal region as the steps progress, and a larger fraction of molecules is located within the optimal region compared to the conventional method. Different from the conventional approach, most of the population shifts toward the optimal region, as clearly shown in the distribution below Figure 4c. From a quantitative perspective, while 40% of the population becomes localized in the local minimum in the conventional method, only about 20% becomes localized when using the R^2^GB-GA. This observation suggests that the R^2^GB-GA converges toward its optimal region more efficiently than the conventional method.

Next, we examined the capacity of the generated ligands to act as potential inhibitors for HSP90 protein. The generated ligands are listed in Table S1. To evaluate whether the generated candidate ligands could function as competitive inhibitors of the HSP90 protein, the binding affinities of the candidate molecules were estimated through molecular docking simulations (Figure 5a). Docking was performed using the GalaxyDock-DL package,^49^ with the docking grid centered on the ATP-binding pocket of HSP90. A cubic grid box with size of 22.5 × 22.5 × 22.5 Å was centered on the ATP-binding pocket, using the coordinates of the bound ATP molecule as the reference point. As shown in Figure 5a, although several ligands from the generative pool exhibited lower binding affinities, a large proportion of the generated molecules were distributed in the region corresponding to high binding affinity values. To verify whether the bound ligands act as competitive inhibitors at the ATP-binding site, we examined the binding poses of the generated ligands with the HSP90 protein. As illustrated in Figure 5b, the ligands exhibiting higher predicted affinities were found to bind in poses consistent with occupation of the ATP binding pocket, whereas those with lower predicted affinities failed to bind properly within the binding site (Figure S2). Furthermore, analysis of protein–ligand interactions (Figure 5c) revealed that high affinity ligands formed a hydrogen bond network with key residues such as Asp93 and Thr184 near the resorcinol moiety, mimicking the hydrogen bond interactions of the adenine ring of ATP.^68,69^ This observation supports the hypothesis that the generated ligands can act as potential inhibitors of HSP90.

**Figure 5:**
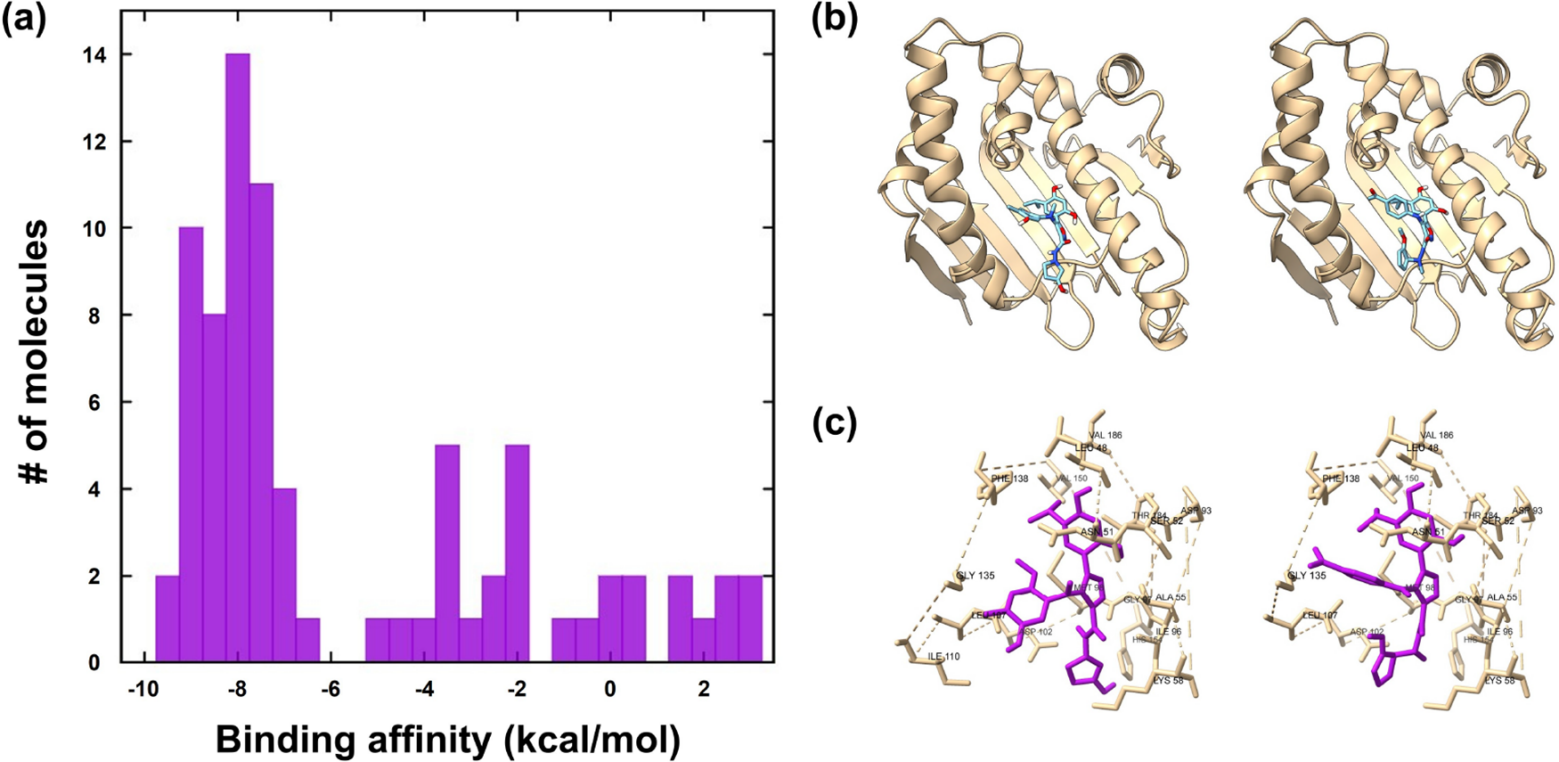
(a) Distribution of binding affinities for ligands generated as HSP90 inhibitors. (b) Cartoon representation of HSP90 in complex with generated ligands. (c) Close-up view of the ligand binding pocket highlighting neighboring residues.

Finally, to assess the practical synthesizability of the proposed candidates, we performed a computational retrosynthetic analysis of the generated molecules. Two candidates from our final generation pool (Compounds 1 and 13) were examined and the resulting retrosynthetic routes are provided in Supporting Information S5. The retrosynthetic routes were constructed by tracing the reactions used to generate the corresponding candidates. We further examined whether an experimentally reported HSP90 inhibitor, which shares a resorcinol motif with Compound 1, could be rediscovered by augmenting the reaction pool. For the reported ligand,^70^ we supplemented the reaction pool with an aromatic bromination reaction used in the literature synthesis that was not present in our reaction pool. With this addition, it could be rediscovered by our generation model (Figure S6). Collectively, these results show that molecules generated by R^2^GB-GA are not only synthetically accessible according to feasibility metrics but also provide plausible retrosynthetic routes.

## 4 Conclusions

In this study, we propose a reaction-driven generative model, named R^2^GB-GA, for the design of synthetically feasible molecular editing in drug discovery. This was achieved by incorporating a predefined reaction database based on actual chemical reactions into the generation process, in contrast to conventional approaches. Our method was validated by comparison with the conventional method using various synthetic feasibility metrics, demonstrating that the R^2^GB-GA generated more synthetically accessible molecules. We also performed a goal-directed benchmark to assess the ability of the R^2^GB-GA to explore the chemical space, and finally designed candidate ligands for potential HSP90 inhibitors using our method, starting from two resorcinol-based scaffolds.

We expect that the R^2^GB-GA will become a promising approach when integrated into active-learning frameworks with experimental validations. This prospect is supported by key features of the proposed generative model. First, our method traces the reactions and reactants used to generate each candidate molecule, thereby enabling experimentalists to reproduce computational results by providing the corresponding reactions and precursors derived from the generative model. It should be noted that the routes obtained with R^2^GB-GA are not guaranteed to be minimal synthetic routes since our framework provides routes obtained by tracing the reactions applied during generation, rather than performing retrosynthetic planning to identify minimal routes. Second, because the reaction pool can be predefined flexibly, it can be adjusted to match the specific initial data set or experimental settings used in molecular design tasks. For example, it is possible to restrict the available reactants or assign higher weights to specific reactions such as prioritizing late-stage functionalization reactions that selectively modify a predefined site. In addition, R2GB-GA can not only mimic late-stage transformations but also explore structural modifications that are difficult to implement at the late stage yet straightforward from a retrosynthetic root perspective. Provided that appropriate building blocks and applicable reactions are available, R^2^GB-GA can, in principle, generate a wide range of desired analogs.

As a next step, we plan to apply the capabilities of our proposed method to designing inhibitors for additional therapeutic targets, in collaboration with experimental studies to advance practical molecular design. We expect that this approach will bridge the gap between theory and experiment and enable more efficient molecular design.

## Supplementary Material



## Data Availability

The source code for the R^2^GB-GA related to HSP90 ligand generation is available on GitHub at https://github.com/hwk-grp/R2GB-GA.

## Notes

Detailed information on HSP90 ligand design (PDF)

## References

[1] Galloway W. R. J. D., Isidro-Llobet A., Spring D. R. (2010). Diversity-Oriented
Synthesis as a Tool for the Discovery of Novel Biologically Active
Small Molecules. Nat. Commun..

[2] Marfavi A., Kavianpour P., Rendina L. M. (2022). Carboranes in Drug Discovery, Chemical
Biology and Molecular Imaging. Nat. Rev. Chem..

[3] Catacutan D. B., Alexander J., Arnold A., Stokes J. M. (2024). Machine Learning
in Preclinical Drug Discovery. Nat. Chem. Biol..

[4] Rose P., Nasim J., Zhu Y. Z. (2024). Editorial:
Novel Compounds from Chemistry
to Druggable Candidates. Front. Chem..

[5] Sanchez S., Boissière C., Cassaignon S., Chanéac C., Durupthy O., Faustini M., Grosso D., Laberty-Robert C., Nicole L., Portehault D., Ribot F., Rozes L., Sassoye C. (2014). Molecular Engineering
of Functional Inorganic and Hybrid
Materials. Chem. Mater..

[6] Liu Y., Zhao T., Ju W., Shi S. (2017). Materials Discovery
and Design Using Machine Learning. J. Materiomics..

[7] Butler K. T., Davies D. W., Cartwright H., Isayev O., Walsh A. (2018). Machine Learning
for Molecular and Materials Science. Nat..

[8] Zheng Y., Yu Z., Zhang S., Kong X., Michaels W., Wang W., Chen G., Liu D., Lai J.-C., Prine N., Zhang W., Nikzad S., Cooper C. B., Zhong D., Mun J. H., Zhang Z., Kang J., Tok J. B.-H., McCulloch I., Qin J. (2021). A Molecular Design Approach towards
Elastic and Multifunctional Polymer Electronics. Nat. Commun..

[9] Vamathevan J., Clark D., Czodrowski P., Dunham I., Ferran E., Lee G., Li B., Madabhushi A., Shah P., Spitzer M., Zhao S. (2019). Applications
of Machine Learning in Drug Discovery and Development. Nat. Rev. Drug Discovery.

[10] Brown N., Ertl P., Lewis R., Luksch T., Reker D., Schneider N. (2020). Artificial
Intelligence in Chemistry and Drug Design. J.
Comput. Aided Mol. Des..

[11] Meyers J., Fabian B., Brown N. (2021). De Novo Molecular Design
and Generative
Models. Drug Discovery Today.

[12] Xia Y., Wang Y., Wang Z., Zhang W. (2024). A Comprehensive Review
of Molecular Optimization in Artificial Intelligence-based Drug Discovery. Quant. Biol..

[13] Gómez-Bombarelli R., Wei J. N., Duvenaud D., Hernández-Lobato J. M., Sánchez-Lengeling B., Sheberla D., Aguilera-Iparraguirre J., Hirzel T. D., Adams R. P., Aspuru-Guzik A. al. e. (2018). Automatic
Chemical Design Using a Data-Driven Continuous Representation of Molecules. ACS Cent. Sci..

[14] Elton D. C., Boukouvalas Z., Fuge M. D., Chung P. W. (2019). Deep Learning
for
Molecular Design—a Review of the State of the Art. Mol. Syst. Des. Eng..

[15] Zeng X., Wang F., Luo Y., Kang S.-g., Tang J., Lightstone F. C., Fang E. F., Cornell W., Nussinov R., Cheng F. (2022). Deep Generative Molecular Design Reshapes Drug Discovery. Cell Rep. Med..

[16] Venkatasubramanian V., Chan K., Caruthers J. M. (1995). Evolutionary
Design of Molecules
with Desired Properties Using the Genetic Algorithm. J. Chem. Inf. Comput. Sci..

[17] Sumita M., Yang X., Ishihara S., Tamura R., Tsuda K. (2018). Hunting for
Organic Molecules with Artificial Intelligence: Molecules Optimized
for Desired Excitation Energies. ACS Cent. Sci..

[18] Reutlinger M., Rodrigues T., Schneider P., Schneider G. (2014). Multi-Objective
Molecular de Novo Design by Adaptive Fragment Prioritization. Angew. Chem., Int. Ed..

[19] Katoch S., Chauhan S. S., Kumar V. (2021). A Review on Genetic Algorithm: Past,
Present, and Future. Multimed. Tools Appl..

[20] Brown N. J., McKay B., Gilardoni F., Gasteiger J. (2004). A Graph-Based
Genetic Algorithm and Its Application to the Multiobjective Evolution
of Median Molecules. J. Chem. Inf. Comput. Sci..

[21] Häse F., Roch L. M., Aspuru-Guzik A. (2019). Next-Generation
Experimentation with
Self-Driving Laboratories. Trends Chem..

[22] Grisoni F., Huisman B. J. H., Button A. L., Moret M., Atz K., Merk D., Schneider G. (2021). Combining
Generative Artificial Intelligence
and On-Chip Synthesis for de Novo Drug Design. Sci. Adv..

[23] Isigkeit L., Hörmann T., Schallmayer E., Scholz K., Lillich F. F., Ehrler J. H. M., Hufnagel B., Büchner J., Marschner J. A., Pabel J., Proschak E., Merk D. (2024). Automated
Design of Multi-Target Ligands by Generative Deep Learning. Nat. Commun..

[24] Tom G., Schmid S. P., Baird S. G., Cao Y., Darvish K., Hao H., Lo S., Pablo-García S., Rajaonson E. M., Skreta M., Yoshikawa N., Corapi S., Akkoc G. D., Strieth-Kalthoff F., Seifrid M., Aspuru-Guzik A. (2024). Self-Driving
Laboratories for Chemistry and Materials Science. Chem. Rev..

[25] Gao W., Coley C. W. (2020). The Synthesizability
of Molecules Proposed by Generative
Models. J. Chem. Inf. Model..

[26] Zhang X., Gao H., Qi Y., Li Y., Wang R. (2025). Generation of Rational
Drug-like Molecular Structures through a Multiple-Objective Reinforcement
Learning Framework. Molecules.

[27] Sun K., Bagni D., Cavanagh J. M., Wang Y., Sawyer J. M., Zhou B., Zhang O., Head-Gordon T., Head-Gordon T. (2025). SynLlama: Generating Synthesizable
Molecules and Their
Analogs with Large Language Models. ACS Cent.
Sci..

[28] Ertl P., Schuffenhauer A. (2009). Estimation
of Synthetic Accessibility Score of Drug-like
Molecules Based on Molecular Complexity and Fragment Contributions. J. Cheminform..

[29] Coley C. W., Rogers L., Green W. H., Jensen K. F. (2018). SCScore: Synthetic
Complexity Learned from a Reaction Corpus. J.
Chem. Inf. Model..

[30] Thakkar A., Chadimová V., Bjerrum E. J., Engkvist O., Reymond J.-L. (2021). Retrosynthetic
Accessibility Score (RAscore) – Rapid Machine Learned Synthesizability
Classification from AI Driven Retrosynthetic Planning. Chem. Sci..

[31] Kim H., Lee K., Kim C., Lim J., Kim W. Y. (2024). DFRscore: deep learning-based
scoring of synthetic complexity with drug-focused retrosynthetic analysis
for high-throughput virtual screening. J. Chem.
Inf. Model..

[32] Kerstjens A., De Winter H. (2022). LEADD: Lamarckian evolutionary algorithm for de novo
drug design. J. Cheminform..

[33] Horwood J., Noutahi E. (2020). Molecular Design in
Synthetically Accessible Chemical
Space via Deep Reinforcement Learning. ACS Omega.

[34] Coley C. W., Rogers L., Green W. H., Jensen K. F. (2017). Computer-Assisted
Retrosynthesis Based on Molecular Similarity. ACS Cent. Sci..

[35] Genheden S., Thakkar A., Chadimová V., Reymond J.-L., Engkvist O., Bjerrum E. (2020). AiZynthFinder: a fast,
robust and flexible open-source
software for retrosynthetic planning. J. Cheminform..

[36] Guo J., Schwaller P. (2025). Directly optimizing for synthesizability in generative
molecular design using retrosynthesis models. Chem. Sci..

[37] Cernak T., Dykstra K. D., Tyagarajan S., Vachal P., Krska S. W. (2016). The medicinal
chemist’s toolbox for late stage functionalization of drug-like
molecules. Chem. Soc. Rev..

[38] Whitesell L., Lindquist S. L. (2005). HSP90 and
the Chaperoning of Cancer. Nat. Rev. Cancer.

[39] Wandinger S. K., Richter K., Buchner J. (2008). The Hsp90
Chaperone Machinery. J. Biol. Chem..

[40] Birbo B., Madu E. E., Madu C. O., Jain A., Lu Y. (2021). Role of HSP90
in Cancer. Int. J. Mol. Sci..

[41] Liang X., Chen R., Wang C., Wang Y., Zhang J. (2024). Targeting
HSP90 for Cancer Therapy: Current Progress and Emerging Prospects. J. Med. Chem..

[42] Zhang Q., Shi B.-F. (2021). Site-Selective Functionalization
of Remote Aliphatic
C–H Bonds via C–H Metallation. Chem. Sci..

[43] Jensen J.
H. (2019). A Graph-Based
Genetic Algorithm and Generative Model/Monte Carlo Tree Search for
the Exploration of Chemical Space. Chem. Sci..

[44] Henault E. S., Rasmussen M. H., Jensen J. H. (2020). Chemical Space Exploration: How Genetic
Algorithms Find the Needle in the Haystack. PeerJ. Phys. Chem..

[45] Mary, H. e. a. datamol-io. 2023; https://datamol.io/.

[46] Rogers D., Hahn M. (2010). Extended-Connectivity Fingerprints. J. Chem.
Inf. Model..

[47] Landrum, G. Open-source cheminformatics software. 2020; https://www.rdkit.org/.

[48] O’Boyle N. M., Banck M., James C. A., Morley C., Vandermeersch T., Hutchison G. R. (2011). Open Babel: An Open Chemical Toolbox. J. Cheminform..

[49] Lee C., Won J., Ryu S., Yang J., Jung N., Park H., Seok C. (2024). GalaxyDock-DL:
Protein–Ligand Docking by Global Optimization
and Neural Network Energy. J. Chem. Theory Comput..

[50] Trott O., Olson A. J. (2009). AutoDock Vina: Improving the Speed
and Accuracy of
Docking with a New Scoring Function, Efficient Optimization, and Multithreading. J. Comput. Chem..

[51] Eberhardt J., Santos-Martins D., Tillack A. F., Forli S. (2021). AutoDock Vina 1.2.0:
New Docking Methods, Expanded Force Field, and Python Bindings. J. Chem. Inf. Model..

[52] Voršilák M., Kolář M., Čmelo I., Svozil D. (2020). SYBA: Bayesian Estimation
of Synthetic Accessibility of Organic Compounds. J. Cheminform..

[53] Mendez D., Gaulton A., Bento A. P., Chambers J., De Veij M., Félix E., Magariños M. P., Mosquera J. F., Mutowo P., Nowotka M., Gordillo-Marañón M., Hunter F., Junco L., Mugumbate G., Rodriguez-Lopez M., Atkinson F., Bosc N., Radoux C. J., Segura-Cabrera A., Hersey A., Leach A. R. (2019). ChEMBL:
Towards
Direct Deposition of Bioassay Data. Nucleic
Acids Res..

[54] Ruddigkeit L., van Deursen R., Blum L. C., Reymond J.-L. (2012). Enumeration of 166
Billion Organic Small Molecules in the Chemical Universe Database
GDB-17. J. Chem. Inf. Model..

[55] Ramakrishnan R., Hartmann M., Tapavicza E., von Lilienfeld O. A. (2015). Electronic
Spectra from TDDFT and Machine Learning in Chemical Space. J. Chem. Phys..

[56] Karageorgis G., Douglas J. J., Howell G. P. (2024). Analysis
of the Change in Molecular
Complexity of Reaction Products in Process Development Activities
at AstraZeneca over Time. Org. Process Res.
Dev..

[57] Brown N., Fiscato M., Segler M. H., Vaucher A. C. (2019). GuacaMol: Benchmarking
Models for de Novo Molecular Design. J. Chem.
Inf. Model..

[58] Post-processed ChEMBL datasets. https://figshare.com/projects/GuacaMol/56639.

[59] Lowe, D. Chemical Reactions from US Patents (1976–Sep2016). 2017; https://figshare.com/articles/dataset/Chemical_reactions_from_US_patents_1976-Sep2016_/5104873.

[60] Chaudhury S., D’Amico T., Blagg B. S. J. (2025). The Hsp90β Isoform: An Attractive
Target for Drug Development. Med. Res. Rev..

[61] Zhang H., Yin X., Zhang X., Zhou M., Xu W., Wei Z., Song C., Han S., Han W. (2022). HSP90AB1 Promotes the
Proliferation, Migration, and Glycolysis of Head and Neck Squamous
Cell Carcinoma. Technol. Cancer Res. Treat..

[62] Lin X., Liu Y.-h., Zhang H.-q., Wu L.-w., Li Q., Deng J., Zhang Q., Yang Y., Zhang C., Li Y.-l., Hu J. (2023). DSCC1 Interacts
with HSP90AB1 and
Promotes the Progression of Lung Adenocarcinoma via Regulating ER
Stress. Cancer Cell Int..

[63] Sharp S. Y., Boxall K., Rowlands M., Prodromou C., Roe S. M., Maloney A., Powers M., Clarke P. A., Box G., Sanderson S., Patterson L., Matthews T. P., Cheung K.-M. J., Ball K., Hayes A., Raynaud F., Marais R., Pearl L., Eccles S., Aherne W., McDonald E., Workman P. (2007). In Vitro Biological Characterization of a Novel, Synthetic
Diaryl Pyrazole Resorcinol Class of Heat Shock Protein 90 Inhibitors. Cancer Res..

[64] Gedgaudas M., Kaziukonytė P., Kairys V., Mickevičiu̅tė A., Zubrienė A., Brukštus A., Matulis D., Kazlauskas E. (2024). Comprehensive
Analysis of Resorcinyl-Imidazole Hsp90 Inhibitor Design. Eur. J. Med. Chem..

[65] Park S. Y., Oh Y. J., Lho Y., Jeong J. H., Liu K.-H., Song J., Kim S.-H., Ha E., Seo Y. H. (2018). Design,
Synthesis, and Biological Evaluation of a Series of Resorcinol-Based
N-benzyl Benzamide Derivatives as Potent Hsp90 Inhibitors. Eur. J. Med. Chem..

[66] Zdrazil B., Félix E., Hunter F., Manners E., Blackshaw J., Corbett S., Veij M. D., Ioannidis H., Méndez D., Mosquera J. F., Magariños M. P., Bosc N., Arcila R., Kizilören T., Gaulton A., Bento A. P., Adasme M. F., Monecke P., Landrum G. A., Leach A. R. (2023). The ChEMBL Database in 2023: A Drug
Discovery Platform Spanning Multiple Bioactivity Data Types and Time
Periods. Nucleic Acids Res..

[67] Maaten L. v. d., Hinton G. (2008). Visualizing data using t-SNE. J. Mach. Learn. Res..

[68] Khandelwal A., Crowley V. M., Blagg B. S. J. (2016). Natural Product Inspired N-Terminal
Hsp90 Inhibitors: From Bench to Bedside?. Med.
Res. Rev..

[69] Khandelwal A., Kent C. N., Balch M., Peng S., Mishra S. J., Deng J., Day V. W., Liu W., Subramanian C., Cohen M. S., Holzbeierlein J. M., Matts R. L., Blagg B. S. J. (2018). Structure-Guided
Design of an Hsp90β N-terminal Isoform-Selective Inhibitor. Nat. Commun..

[70] Kang J., Pagire H. S., Kang D., Song Y. H., Lee I. K., Lee K. T., Park C.-J., Ahn J. H., Kim J. (2020). Structural
basis for the inhibition of PDK2 by novel ATP- and lipoyl-binding
site targeting compounds. Biochem. Biophys.
Res. Commun..

