# Supplementary material for: Enabling Synthetically Feasible Molecular Editing in Drug Discovery via Reaction-Regulated Graph-Based Genetic Algorithms
Source: JACS Au. 2026 Mar 23;6(4):2506–15. doi: 10.1021/jacsau.6c00094 (PMC13126195; doi:10.1021/jacsau.6c00094)
Supplement: Supplementary file 1 [file au6c00094_si_001.pdf]

Supporting Information: Enabling Synthetically Feasible Molecular Editing in Drug Discovery via Reaction-Regulated Graph-Based Genetic Algorithms

Sung Wook Moon,[†] Se Hwan Ahn,[†] Jin Hee Ahn,[*,†,‡] and Hyun Woo Kim[*,†]

†*Department of Chemistry, Gwangju Institute of Science and Technology, Gwangju, 61005, Republic of Korea*

‡*JD Bioscience Inc., Gwangju 61011, Republic of Korea*

E-mail: jhahn@gist.ac.kr; hwk@gist.ac.kr

Contents

S1. Ligand design for HSP90 inhibition 3

S2. Benchmarking synthetic feasibility without RAscore-overlapping reactions . . 6

S3. Construction and evaluation of downsampled reaction pools for the rediscovery task 7

S4. Statistics of reaction usage frequencies and their effects on HSP90 inhibitor generation 8

S5. Retrosynthetic routes for two HSP90 candidate inhibitors 10

References **12**

S1. Ligand design for HSP90 inhibition

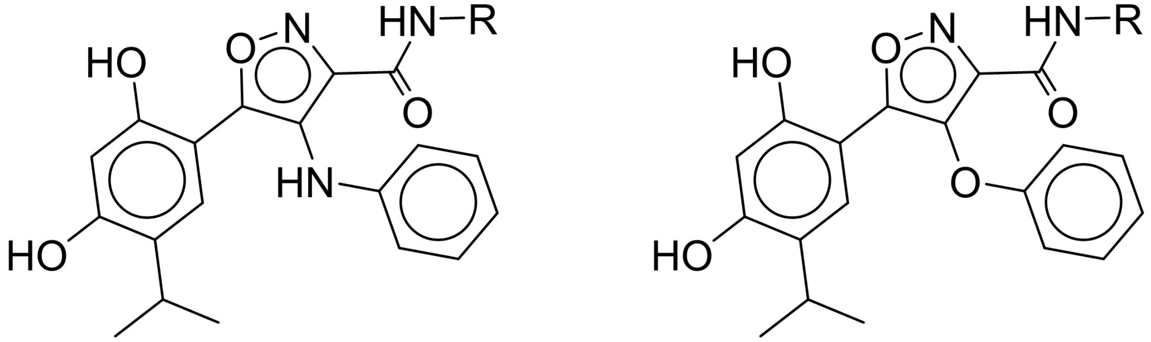

Figure S1: Basic scaffolds for resorcinol-based HSP90 inhibitor design.

Table S1: Ligands for HSP90 inhibition along with their predicted IC_{50} values in nM

SMILES	predicted
CNC(=O)Oc1cc(OC)ccc1N(C(=S)NC)c1c(C(=O)NC)noc1-c1cc(C(C)C)c(O)cc1O	24.52628
CNC(=O)c1noc(-c2cc(C(C)C)c(O)cc2O)c1N(C)c1cc(C(C)C)c2oc(=O)cc(C)c2c1OC	23.14434
COCCNC(=O)c1noc(-c2cc(C(C)C)c(O)cc2O)c1N(C(C)=O)c1cccc2oc(=O)cc(C)c12	24.31256
COCCN(c1cc(C)c(O)cc1OC)c1c(C(=O)NCO)noc1-c1cc(C(C)C)c(O)cc1O	23.20316
COc1cc(C)c(O)cc1N(C)c1c(C(=O)NN2C=C(O)CC2)noc1-c1cc(C(C)C)c(O)cc1O	24.56107
CNC(=O)c1noc(-c2cc(C(C)C)c(O)cc2O)c1N(C)c1cc(C(C)C)c(OC)cc1O	24.79936
CNC(=O)NC(=O)c1noc(-c2cc(C(C)C)c(O)cc2O)c1N(C)c1cc(O)c(C)cc1OC	24.59772
COCCNC(=O)c1noc(-c2cc(C(C)C)c(O)cc2O)c1N(C(=O)CO)c1ccc(C)cc1	24.38601
CNNC(=O)c1noc(-c2cc(C(C)C)c(O)cc2O)c1N(NO)c1c(O)cc(OC)cc1OC	24.63657
CCN(c1ccc2oc(=O)cc(C)c2c1)c1c(C(=O)NCCOC)noc1-c1cc(C(C)C)c(O)cc1O	23.58820
CNC(=O)c1noc(-c2cc(C(C)C)c(O)cc2O)c1Nc1ccc(C(=O)NC(=O)NCCOC)cc1	22.59709
CNC(=O)c1noc(-c2cc(C(C)C)c(O)cc2O)c1N(O)c1c(OC)cc(O)c2oc(=O)cc(C)c12	24.83211
CNC(=O)c1noc(-c2cc(C(C)C)c(O)cc2O)c1N(c1c(C)cc(OC)c2oc(=O)cc(C)c12)C(C)C	23.47525
CNC(=O)c1noc(-c2cc(C(C)C)c(O)cc2O)c1N(C)c1cc(Nc2ccc(OC)cc2)c(O)cc1OC	24.33653
CNC(=O)Oc1cc(O)c(C(=O)NCCOC)cc1N(C)c1c(C(=O)NC)noc1-c1cc(C(C)C)c(O)cc1O	22.91466
CNC(=O)N(c1cc(C)c(OC)cc1OC(C)=O)c1c(C(=O)NCCOO)noc1-c1cc(C(C)C)c(O)cc1O	24.80414
CNC(=O)c1noc(-c2cc(C(C)C)c(O)cc2O)c1N(C)c1cc(SC)c(-n2nncc2C)c(OC)c1OC	24.01848
CNC(=O)c1noc(-c2cc(C(C)C)c(O)cc2O)c1N(C)c1cc(-c2ccccc2)cc(C(C)C)c1OC	24.68563
COc1cc(O)cc(O)c1N(CCNN1CCOCC1)c1c(C(=O)NCO)noc1-c1cc(C(C)C)c(O)cc1O	23.91162
COC1=CC=CC1N(C)NC(=O)c1noc(-c2cc(C(C)C)c(O)cc2O)c1Nc1ccc(C(C)=O)cc1	24.60943
CNNC(=O)c1noc(-c2cc(C(C)C)c(O)cc2O)c1N(NO)c1c(OC)cc(OO)c2c(C)cc(=O)oc12	23.57206
COc1cc(O)cc(O)c1-c1cccc(Nc2c(C(=O)N(O)C(C)=O)noc2-c2cc(C(C)C)c(O)cc2O)c1	24.36480
COCCNC(=O)c1noc(-c2cc(C(C)C)c(O)cc2O)c1N(O)c1cc(C(C)C)c(O)c2c(C)cc(=O)oc12	24.81065
COc1ccc(Br)cc1N(C)c1c(C(=O)NC2=CC(C(C)C)=C(O)C2)noc1-c1cc(C(C)C)c(O)cc1O	24.48923
CNC(=O)CN(c1ccc(C(=O)CO)cc1)c1c(C(=O)NCCOC)noc1-c1cc(C(C)C)c(O)cc1O	23.31028
CNC(=O)N(c1cc(O)c(OC)cc1OC)c1c(C(=O)NCCOC)noc1-c1cc(C(C)C)c(O)cc1O	23.68901
CNC(C)c1cc(O)c(O)cc1N(c1c(C(=O)NCCOC)noc1-c1cc(C(C)C)c(O)cc1O)S(C)(=O)=O	23.74942
CNC(=O)c1noc(-c2cc(C(C)C)c(O)cc2O)c1N(C)c1cc(C(=O)NCCOC)c(OC)cc1OC	21.77392
CNC(=O)c1noc(-c2cc(C(C)C)c(O)cc2O)c1N(c1ccc(OC)c2oc(=O)cc(C)c12)C(C)C	23.58452
COCCN(c1c(OC(C)=O)cc(O)cc1C(=O)OC)c1c(C(=O)NCO)noc1-c1cc(C(C)C)c(O)cc1O	22.90805
CCN(c1cc(C)c(O)cc1CCOC)c1c(C(=O)NC)noc1-c1cc(C(C)C)c(O)cc1O	24.48873
CNC(=O)c1noc(-c2cc(C(C)C)c(O)cc2O)c1N(C(=O)OC)c1ccc(C(=O)NC(=O)NCCOO)cc1	23.52816
CNC(=O)c1noc(-c2cc(C(C)C)c(O)cc2O)c1N(C(=S)NC)c1cc(C)c(OC)cc1Cl	22.64333
COCCNC(=O)c1noc(-c2cc(C(C)C)c(O)cc2O)c1N(CO)c1ccc(C(=O)CC(C)=O)cc1	22.46389
CNC(=O)N(c1ccc(C(=O)NC(=O)NCCOC)cc1)c1c(C(=O)NO)noc1-c1cc(C(C)C)c(O)cc1O	23.28048
C=C(C)c1cc(-c2c(O)cc(Nc3c(C(=O)NC)noc3-c3cc(C(C)C)c(O)cc3O)cc2OC)c(O)cc1O	24.58043
CNC(=O)c1noc(-c2cc(C(C)C)c(O)cc2O)c1N(C(=O)NC)c1cc(OC)c(-c2cccc(Br)c2)cc1O	24.08639
C=C(c1cc(N(C)c2c(C(=O)NCCOC)noc2-c2cc(C(C)C)c(O)cc2O)c(OC)cc1O)C(C)C	20.22461
COCCNC(=O)c1noc(-c2cc(C(C)C)c(O)cc2O)c1N(C(=O)CO)c1ccc(O)c2c(C)cc(=O)oc12	24.76142
COCCNC(=O)c1noc(-c2cc(C(C)C)c(O)cc2O)c1N(O)c1ccccc1OC	23.72437
CNC(C)c1c(O)cc(OC)cc1N(NC)c1c(C(=O)NCCOC)noc1-c1cc(C(C)C)c(O)cc1O	24.05038
COCCNC(=O)c1noc(-c2cc(C(C)C)c(O)cc2O)c1N(C)c1cc(O)c(OC)cc1OC	21.98281
COCCNC(=O)c1noc(-c2cc(C(C)C)c(O)cc2O)c1Nc1cc(C(C)C)c(O)cc1O	24.62977
CCN(c1cc(C)c(O)cc1OC(=O)NC)c1c(C(=O)NCCOC)noc1-c1cc(C(C)C)c(O)cc1O	24.38568
CNC(=O)c1noc(-c2cc(C(C)C)c(O)cc2O)c1N(NC)c1cc(C)c(OC)cc1Cl	23.25883
COCCNC(=O)c1noc(-c2cc(C(C)C)c(O)cc2O)c1Nc1cc(O)c(OC(=O)COC)c(OC(C)=O)c1	23.90040
CCCNC(=O)c1ccc(N(c2c(C(=O)NC)noc2-c2cc(C(C)C)c(O)cc2O)S(C)(=O)=O)cc1	23.28913
COCCNC(=O)c1noc(-c2cc(C(C)C)c(O)cc2O)c1N(C)c1cc(C)c2oc(=O)cc(C)c2c1OC	22.41851
C=C(c1cc(N(O)c2c(C(=O)NC)noc2-c2cc(C(C)C)c(O)cc2O)c(OC)cc1O)C(C)C	24.63402
CNC(=S)N(c1ccc(OC)cc1C(=O)OC)c1c(C(=O)NCCOC)noc1-c1cc(C(C)C)c(O)cc1O	23.34813
CNC(=O)c1noc(-c2cc(C(C)C)c(O)cc2O)c1N(C)c1cc(CCO)cc(C(C)C)c1OC	24.13686
CNC(=O)CN(c1cc(Br)ccc1OC)c1c(C(=O)NCCOC)noc1-c1cc(C(C)C)c(O)cc1O	22.43104
CNC(=O)ON(c1ccc(C(=O)CO)cc1)c1c(C(=O)NCCOC)noc1-c1cc(C(C)C)c(O)cc1O	24.04994
CNC(=O)Oc1cc(O)cc(OC)c1N(CCOC)c1c(C(=O)NCO)noc1-c1cc(C(C)C)c(O)cc1O	24.48168
COCCNC(=O)c1noc(-c2cc(C(C)C)c(O)cc2O)c1N(c1ccc(C)cc1)C(C)C	22.78140
COCCNC(=O)c1noc(-c2cc(C(C)C)c(O)cc2O)c1N(CCOC)c1ccc(C)cc1	24.04223
CCC(=O)Oc1cc(OC)c(N(CC)c2c(C(=O)NCCOC)noc2-c2cc(C(C)C)c(O)cc2O)cc1C	23.79939
CNC(=O)c1noc(-c2cc(C(C)C)c(O)cc2O)c1Nc1cc(OC)c(C)c(OC)c1	23.44156
CNC(=O)OCCNC(=O)c1noc(-c2cc(C(C)C)c(O)cc2O)c1N(C)c1cc(Br)ccc1OC	23.99927
CNNC(=O)c1noc(-c2cc(C(C)C)c(O)cc2O)c1N(NO)c1cc(C(C)C)c2oc(=O)cc(C)c2c1OC	24.10362
COc1c(O)cc(O)cc1N(C)c1c(C(=O)NN2C=C(O)CC2)noc1-c1cc(C(C)C)c(O)cc1O	24.50749
COc1cc(N(C2=C(O)C=C(O)C2)c2c(C(=O)NO)noc2-c2cc(C(C)C)c(O)cc2O)c(OC)cc1C	24.40373

SMILES	predicted
COCCNC(=O)c1noc(-c2cc(C(C)C)c(O)cc2O)c1Nc1cc(O)cc(C(C)C)c1	24.53001
CCN(c1cc(C(C)C)cc(-n2nncc2C)c1)c1c(C(=O)NCCOC)noc1-c1cc(C(C)C)c(O)cc1O	22.34786
CNC(=O)c1noc(-c2cc(C(C)C)c(O)cc2O)c1N(C)c1cc(Br)c2oc(=O)cc(O)c2c1OC	24.21023
CNC(=O)c1cc(O)cc(CCOC)c1N(CCOC)c1c(C(=O)NCO)noc1-c1cc(C(C)C)c(O)cc1O	23.75849
CNNC(=O)c1noc(-c2cc(C(C)C)c(O)cc2O)c1N(NO)c1cc(O)c2oc(=O)cc(Br)c2c1OC	24.74317
CNC(=O)CN(c1cc(C)ccc1C(C)C)c1c(C(=O)NCCOC)noc1-c1cc(C(C)C)c(O)cc1O	24.49755
CNC(=O)c1noc(-c2cc(C(C)C)c(O)cc2O)c1N(C)c1cc(C(C)C)c(OC)cc1-c1ccc(C)cc1	24.36914
COCCNC(=O)c1noc(-c2cc(C(C)C)c(O)cc2O)c1N(CO)c1cccc(O)c1	22.65392
C=C(c1cc(N(NCCOC)c2c(C(=O)NC)noc2-c2cc(C(C)C)c(O)cc2O)c(OC)cc1O)C(C)C	21.12106
CNC(=O)c1noc(-c2cc(C(C)C)c(O)cc2O)c1N(C)c1c(OC)cc(O)c(OC)c1OC	24.82540
CNC(=O)c1noc(-c2cc(C(C)C)c(O)cc2O)c1N(C(=S)NC)c1cc(OC)c(OC)c(OC)c1	23.60383
COCCNC(=O)c1cccc(Nc2c(C(=O)N(O)C(C)=O)noc2-c2cc(C(C)C)c(O)cc2O)c1	19.58150
COCCNC(=O)c1noc(-c2cc(C(C)C)c(O)cc2O)c1Nc1cc(O)cc(NN2C=C(O)CC2)c1	23.92097
COc1ccc(C)cc1N(C)c1c(C(=O)NCCOC(C)C)noc1-c1cc(C(C)C)c(O)cc1O	23.85430
CNC(=O)c1noc(-c2cc(C(C)C)c(O)cc2O)c1N(CCO)c1ccc(OC)cc1	24.84262
CNC(=O)c1cc(OC)cc(C(C)C)c1N(CCOC)c1c(C(=O)NCO)noc1-c1cc(C(C)C)c(O)cc1O	23.72368
COCCc1cc(O)cc(OC)c1N(CCOC)c1c(C(=O)NCOC)noc1-c1cc(C(C)C)c(O)cc1O	24.42010
C=C(C)c1cc(OC)c(N(CO)c2c(C(=O)NCCOC(=O)NC)noc2-c2cc(C(C)C)c(O)cc2O)cc1C	21.94962

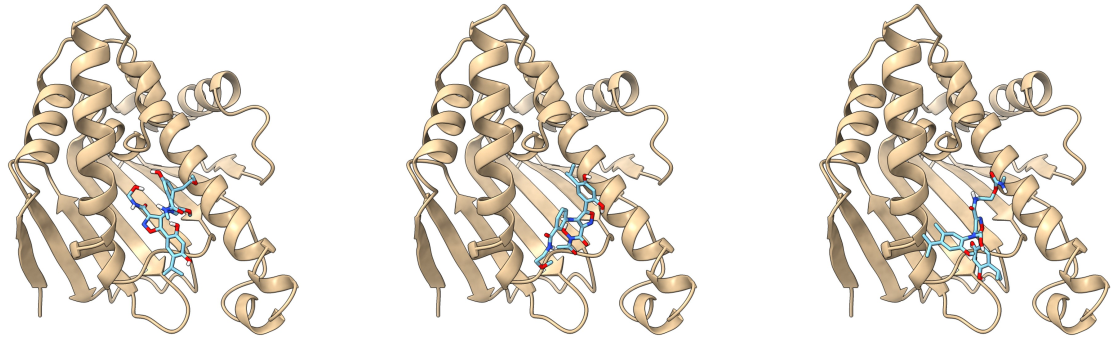

Figure S2: Cartoon representations of the HSP90-ligand complexes showing binding poses of low affinity ligands

S2. Benchmarking synthetic feasibility without RAscore-overlapping reactions

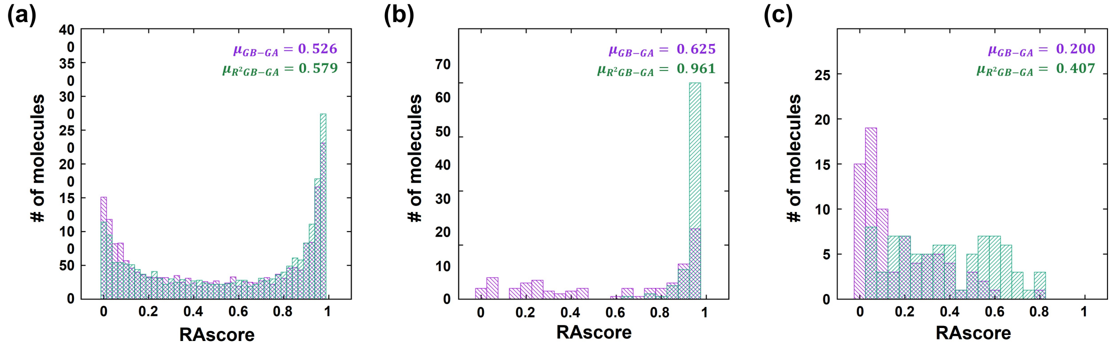

Figure S3: Comparison of RAscore for molecules generated using the conventional GB-GA and R^2GB-GA using a reaction template pool with the reactions associated with the RAscore excluded. (a) Generation of molecules from the ChEMBL database, (b) design of molecules based on the QM8 dataset, and (c) ligand design for the HSP90 inhibitor. Distributions for the conventional GB-GA (purple) and R^2GB-GA (green) are depicted.

S3. Construction and evaluation of downsampled reaction pools for the rediscovery task

Downsampled reaction pools were constructed by randomly sampling subsets from the full reaction pool (N = 362). Six subsets were generated, containing 50, 100, 150, 200, 250, and 300 reactions, respectively. For each subset, we ran 100 independent rediscovery trajectories for up to 100 optimization steps using the same settings as in the main text. The rediscovery target was troglitazone, and performance was evaluated using the average number of steps required to rediscover the target, denoted as $\bar{n}$, for each reaction pool size.

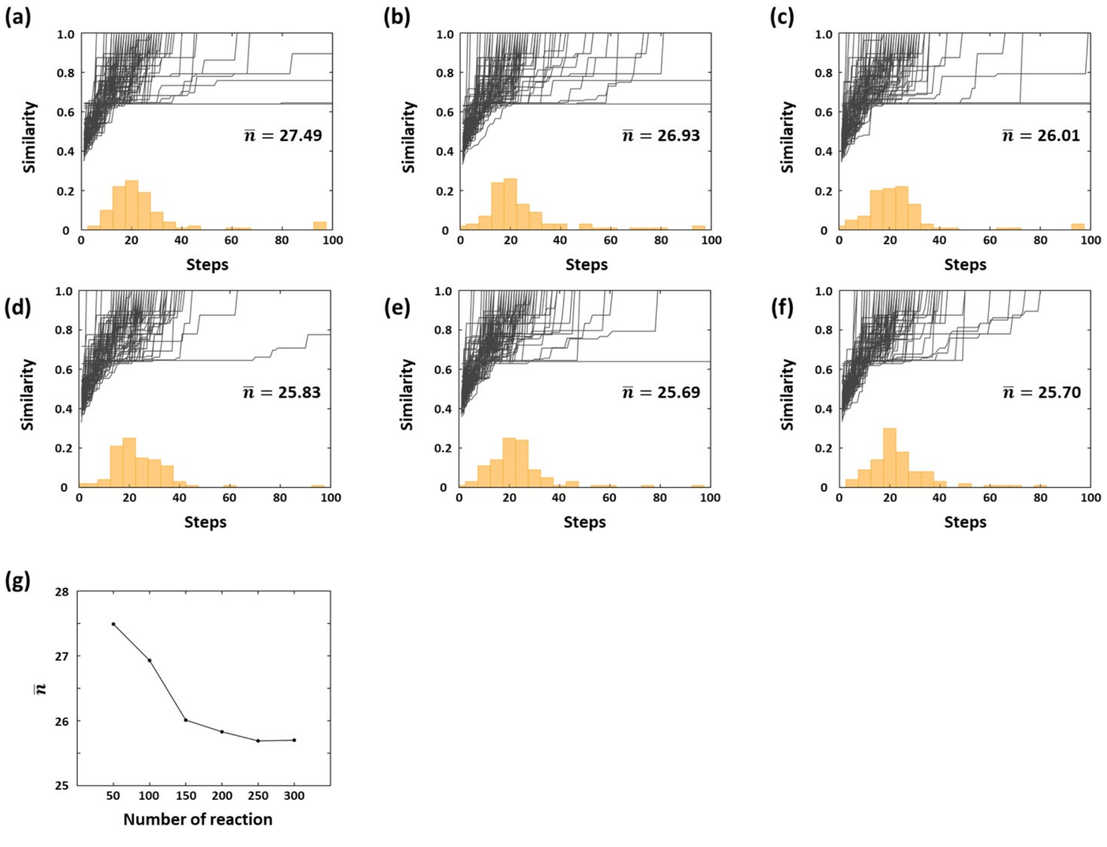

Figure S4: Optimization trajectories of the maximum Tanimoto similarity to troglitazone as a function of optimization step for R^2GB-GA using different sizes of reaction pools. The number of reaction pool sizes are (a) 50, (b) 100, (c) 150, (d) 200, (e) 250, and (f) 300. Gray traces denote individual trajectories, and the orange histograms show the distribution of the number of steps to find the target molecule. The value $\bar{n}$ in each panel denotes the mean number of steps to find the target. (g) Dependence of $\bar{n}$ on the reaction pool size.

S4. Statistics of reaction usage frequencies and their effects on HSP90 inhibitor generation

Table S2: Frequencies of the top 20 most frequently observed reactions in the datamol reaction database.[1] The reaction number provided in the datamol file, the reaction tag in the same file, subtype, and the corresponding count are reported. The total number of occurrences is 1,018,133.

Reaction number	Reaction tag	Subtype	Count
16	`amide_coupling-1`	Amide formation	175821
55	`ester-hydrolysis-1`	Ester hydrolysis	122546
20	`amination-1`	Buchwald–Hartwig amination	115147
113	`suzuki`	Suzuki–Miyaura coupling	111078
17	`amide_coupling-2`	Amide formation	57391
60	`ether-2`	Williamson etherification	56049
14	`alkylation-1`	N-alkylation of amines	52089
10	`BOC-deprotection`	Boc deprotection	43369
109	`sulfonamide-1`	Sulfonamide formation	40062
15	`alkylation-2`	N-alkylation of heterocycles	38566
58	`esterification-2`	Esterification	23883
11	`BOC-protection`	Boc protection	20578
37	`castro-stephens-2`	Castro–Stephens coupling	17011
59	`ether-1`	Ullmann aryl ether coupling	15165
74	`mitsunobu-1`	Mitsunobu substitution	15041
122	`thioether-2`	Thioether formation	14710
79	`miyaura-borylation`	Miyaura borylation	10594
56	`ester-hydrolysis-2`	Ester hydrolysis	8816
57	`esterification-1`	Esterification	7864
107	`stille`	Stille coupling	7560

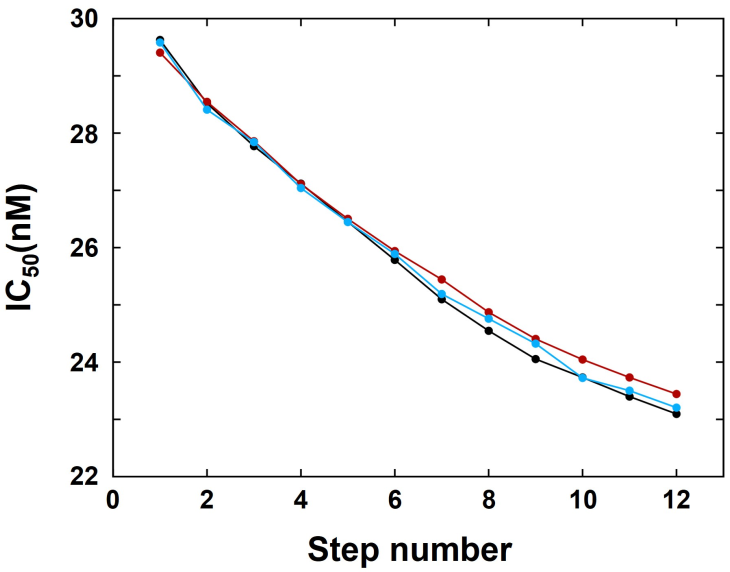

Figure S5: Mean predicted IC_{50} as a function of generation step for HSP90 inhibitor candidate generation using three reaction pools: the full pool (black), a size-matched control pool with 10 reactions removed at random (blue), and a pool excluding the 10 most frequent reactions (red).

S5. Retrosynthetic routes for two HSP90 candidate inhibitors

Scheme S1. Retrosynthetic Analysis of Compound 1[a]

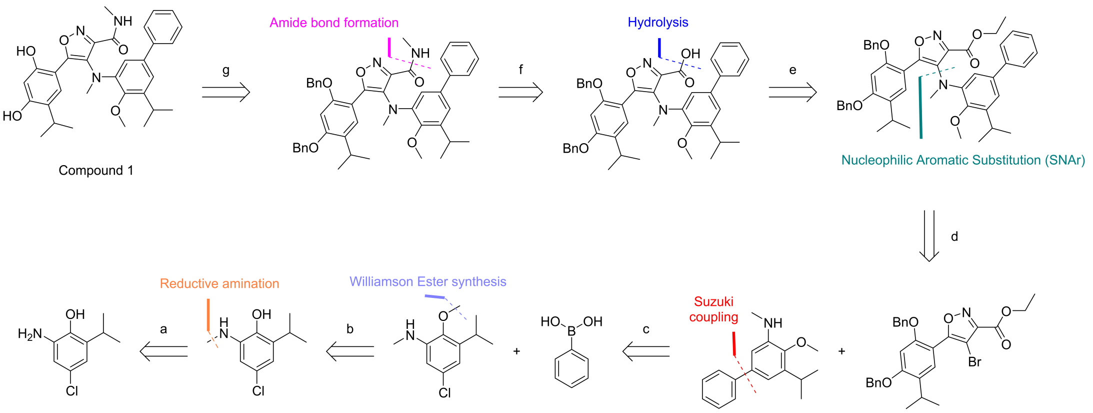

[a]Reagents and conditions: (a) $NaB(OAc)_3H$, AcOH, formaldehyde, rt, 16 h; (b) K_2CO_3, MeI, acetone, reflux, 16 h; (c) $Pd(P(t\text{Bu})_3)_2$, K_3PO_4, 170 °C, 1 h; (d) Cs_2CO_3, ACN, reflux, 16 h; (e) NaOH, THF, H_2O, rt, 24 h; (f) EDCI, HOBt, DIPEA, amine, DCM, rt, 24 h; (g) BCl_3, DCM, rt, 10 min.

Scheme S2. Retrosynthetic Analysis of Compound 13[b]

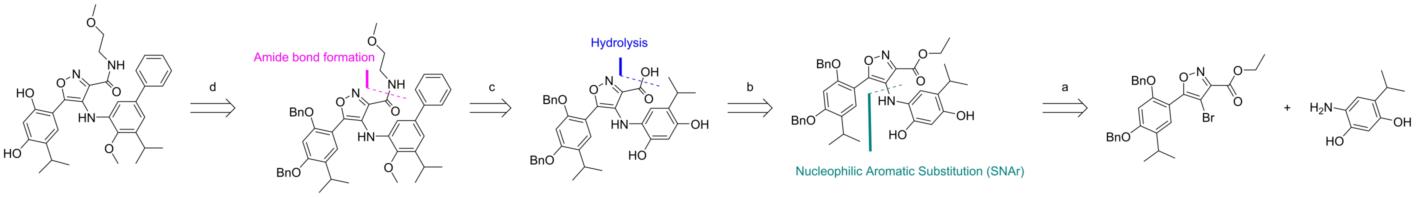

[b]Reagents and conditions: (a) Cs_2CO_3, ACN, reflux, 16 h; (b) NaOH, THF, H_2O, rt, 24 h; (c) EDCI, HOBt, DIPEA, amine, DCM, rt, 24 h; (d) BCl_3, DCM, rt, 10 min.

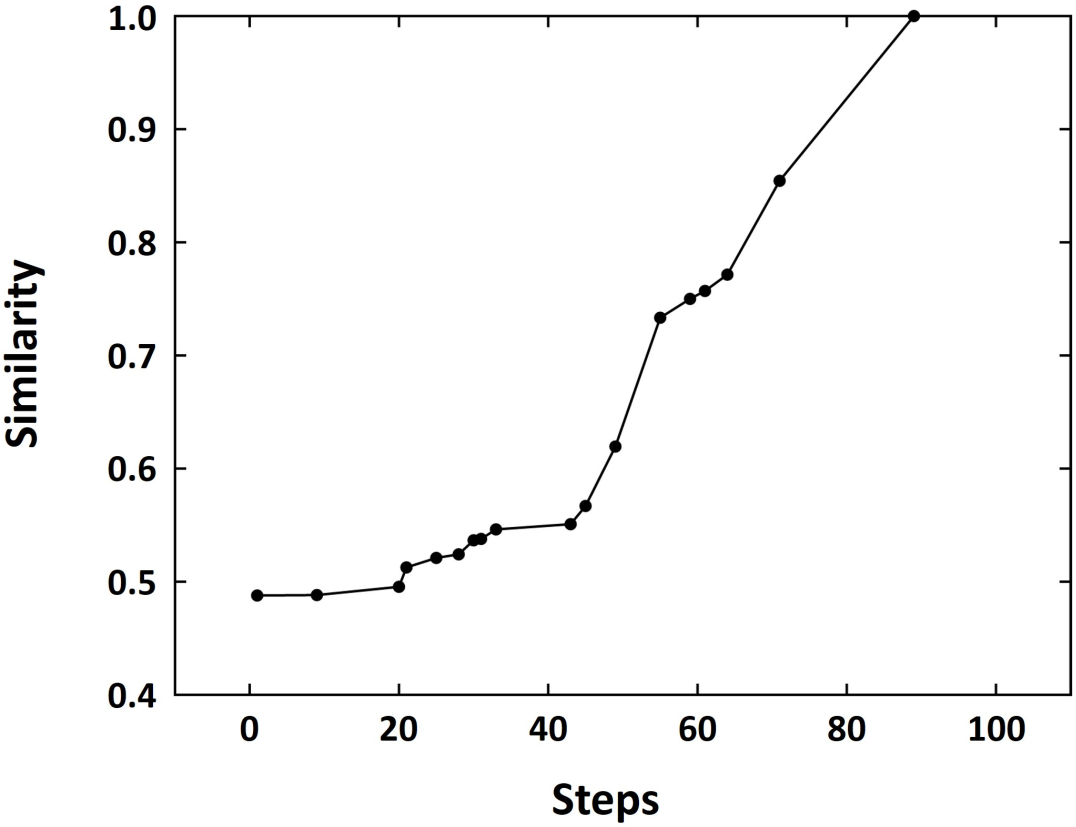

Figure S6: Maximum Tanimoto similarity to GM67520 over generation steps. At each step, the maximum similarity is computed over the current generation pool, and points indicate steps at which the maximum similarity in molecule is updated.

References

(1) Mary, H. e. a. datamol-io. 2023; `https://datamol.io/`.
